# Using Finite Element Models to Assess Spinal Cord Biomechanics after Cervical Laminoplasty for Degenerative Cervical Myelopathy

**DOI:** 10.3390/diagnostics14141497

**Published:** 2024-07-12

**Authors:** Mahmudur Rahman, Peter Palmer, Balaji Harinathan, Karthik Banurekha Devaraj, Narayan Yoganandan, Aditya Vedantam

**Affiliations:** Department of Neurosurgery, Medical College of Wisconsin, Milwaukee, WI 53226, USA

**Keywords:** finite element model, cervical laminoplasty, degenerative cervical myelopathy, cervical kyphosis

## Abstract

Cervical laminoplasty is an established motion-preserving procedure for degenerative cervical myelopathy (DCM). However, patients with pre-existing cervical kyphosis often experience inferior outcomes compared to those with straight or lordotic spines. Limited dorsal spinal cord shift in kyphotic spines post-decompression and increased spinal cord tension may contribute to poor neurological recovery and spinal cord injury. This study aims to quantify the biomechanical impact of cervical sagittal alignment on spinal cord stress and strain post-laminoplasty using a validated 3D finite element model of the C2–T1 spine. Three models were created based on the C2–C7 Cobb angle: lordosis (20 degrees), straight (0 degrees), and kyphosis (−9 degrees). Open-door laminoplasty was simulated at C4, C5, and C6 levels, followed by physiological neck flexion and extension. The results showed that spinal cord stress and strain were highest in kyphotic curvature compared to straight and lordotic curvatures across all cervical segments, despite similar segmental ROM. In flexion, kyphotic spines exhibited 103.3% higher stress and 128.9% higher strain than lordotic spines and 16.7% higher stress and 26.8% higher strain than straight spines. In extension, kyphotic spines showed 135.4% higher stress and 241.7% higher strain than lordotic spines and 21.5% higher stress and 43.2% higher strain than straight spines. The study shows that cervical kyphosis leads to increased spinal cord stress and strain post-laminoplasty, underscoring the need to address sagittal alignment in addition to decompression for optimal patient outcomes.

## 1. Introduction

Cervical laminoplasty is an effective motion-preserving intervention for degenerative cervical myelopathy (DCM) [1,2]. However, cervical kyphosis can adversely impact the neurological outcome for patients undergoing cervical laminoplasty. DCM patients with pre-existing cervical kyphosis show inferior outcomes after cervical laminoplasty as compared to those with straight or lordotic spines [3]. Additionally, the development of kyphosis after laminoplasty also contributes to neurological worsening. Although kyphosis is known to have a negative impact on patients undergoing laminoplasty for DCM, the biomechanical basis for this effect has not been evaluated.

Kyphotic cervical spines show limited dorsal spinal cord shift after laminoplasty, which can explain impaired neurological recovery; however, adverse spinal cord tension can also contribute to ongoing spinal cord damage. Spinal cord stress and strain is known to contribute to spinal cord damage in DCM [4]. Since laminoplasty is a motion-preserving procedure, changes in spinal cord biomechanics with neck motion are expected to further exacerbate adverse spinal cord tension. However, the relationship between spinal cord biomechanics and sagittal alignment after laminoplasty is not well characterized. Determining the effect of sagittal alignment on spinal cord stress and strain after laminoplasty is necessary to improve our understanding of the effects of sagittal alignment on outcomes after laminoplasty.

Finite element modeling (FEM) can quantify the spinal cord stress and strain of the cervical spinal cord. In addition, spinal cord biomechanics can be measured for different sagittal alignments as well as for different surgical procedures [1]. Using human tissue material properties, the FEM approach is able to closely simulate the responses of the human spinal cord for various loading conditions, including physiological neck flexion and extension [1,5,6,7]. Since direct measurements of spinal cord stress and strain are not feasible in humans, FEM techniques offer a unique opportunity to quantify spinal cord biomechanics for different physiological and surgical conditions.

This study aims to quantify the biomechanical impact of cervical sagittal alignment on spinal cord stress and strain after cervical laminoplasty. Since post-operative kyphosis is associated with worse neurological outcomes, we hypothesize that kyphotic alignment results in higher spinal cord stress and strain compared to lordotic and straight alignments. Using a validated FEM of the C2–T1 spine and spinal cord, we will simulate open-door laminoplasty at the C4–C6 and examine the effects of kyphosis, straight, and lordotic curvatures on spinal cord biomechanics. Our goal is to evaluate the biomechanical basis for how different alignments influence spinal cord tension, which will assist in predicting the effects of laminoplasty on outcomes for DCM.

## 2. Materials and Methods

### 2.1. Finite Element Model

In our prior research, we established the material properties for our FEM of the C2–T1 cervical spine using a consistent methodology [8,9]. This involved modeling the intervertebral disk with hexahedral elements for the nucleus and annulus ground and quadrilateral elements for the annulus fibers. The vertebral structure’s cancellous and cortical bones were represented by hexahedral and quadrilateral elements, respectively, made from isotropic linear elastic materials. Endplates, at 0.2 mm thickness, were also depicted using quadrilateral elements. The model incorporated detailed representations of five primary ligament groups: anterior longitudinal ligament (ALL), posterior longitudinal ligament (PLL), interspinous ligament (ISL), ligamentum flavum (LF), and capsular ligament (CL), using quadrilateral membrane elements. The anisotropic behavior of annulus fibrosus was captured using a nonlinear, orthotropic material model, with fibers oriented differently in the anterior and posterior regions. The anterior annular fibers were arranged in a crisscross pattern, contrasting with the vertical alignment of the posterior fibers, to reflect the annulus’s orthotropic nature. This model also included a sophisticated representation of the annulus fibrosus fiber layers, with distinct stress–strain curves for each layer, acknowledging the discontinuity between anterior and posterior fibers at the uncovertebral joints. Materials for the annulus ground and nucleus pulposus were modeled as hill foam and viscoelastic fluid, respectively. The comprehensive mesh comprised 44,799 hexahedral and 35,679 quadrilateral elements. The FEM included the spinal cord, cerebrospinal fluid, pia mater, dura mater, and denticulate ligaments using the material properties listed in previous studies [5]. This FEM was developed in the Department of Neurosurgery at the Medical College of Wisconsin.

### 2.2. FEM Validation

**Figure 1:**
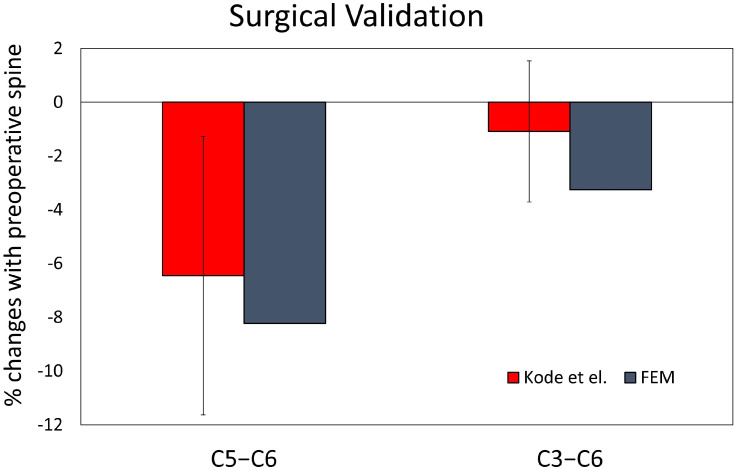
Bar graph showing percent changes in C2–T1 motion for FEM models of C5–C6, and C3–C6 laminoplasty were within the mean ±1 standard deviation from previously published cadaver studies [11].

The generic spine FEM was validated under sagittal bending by comparing the flexion–extension responses from human cadaver cervical columns using 13 subjects with a mean age of 33 years and applying 2 Nm of moment loading to the spinal column [10]. The model-predicted range of motions at all segmental levels for both flexion and extension loading was within the mean ±1 standard deviation of the data from the experiments. For validation of the laminoplasty FEM, predicted percentage changes in C2–T1 motion were noted to be within ±1 standard deviation from cadaver laminoplasty experiments for flexion–extension loading (Figure 1) [11].

### 2.3. Cervical Sagittal Alignment

**Figure 2:**
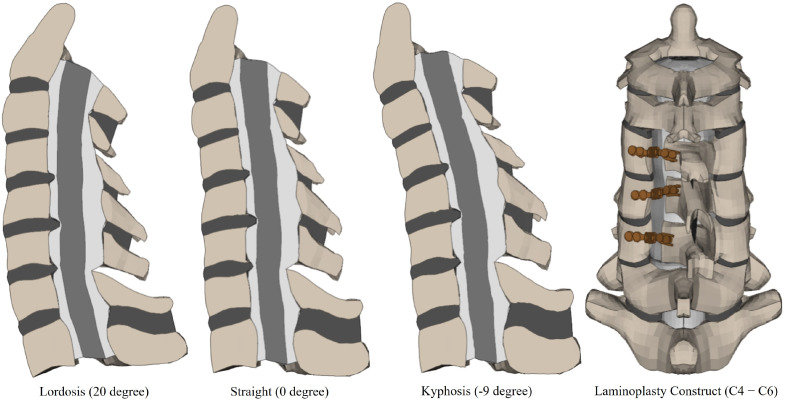
Finite element models of cervical spine and spinal cord after laminoplasty.

A mesh morphing technique was used to modify the sagittal alignment, creating models with varying curvatures from a baseline lordotic model. Utilizing a block-based mapping approach, a unique morphing block was constructed for each functional spinal unit, encompassing the spinal cord. This method enabled the control points, represented by the vertices of these blocks, to guide the transformation of finite element nodes to their new positions through a process of interpolation based on the spatial adjustments of these control points. Starting with a baseline lordotic model characterized by a C2–C7 Cobb angle of 20°, the morphing process produced two additional models: one kyphotic with a Cobb angle of −9° and another with a straight curvature with a Cobb angle of 0° (Figure 2). These angles were chosen based on the prior literature and established norms for cervical spine alignment [12,13]. The morphing parameters were defined to include all components of the functional spinal units. The mesh morphing was executed using the advanced features of the FE preprocessor ANSA^®^ v22.1.0 (BETA CAE Systems, Farmington Hills, MI, USA), which facilitated the intricate morphing process.

### 2.4. Modification of Generic FEM

The intact model was adapted to represent a DCM scenario by introducing a disc bulge in the anterior–posterior direction, resulting in a 44% compression of the spinal cord at C5–C6. This modification also involved altering the intervertebral disc properties to a grade 2 degeneration at C4–C5, C5–C6, and C6–C7 [14]. This grading indicates that the disc’s nucleus has been replaced with a ground substance typical of the annulus, and the thickness of the annulus fibrosus has been reduced to 85% of its original dimension.

### 2.5. Simulation of Laminoplasty in FEM

The laminoplasty intervention was performed on all three models. The surgical simulation involved the formation of a full-thickness trough across the laminae of the C4, C5, and C6 vertebrae at the intersection with the lateral mass, simulating the surgical intervention on one side of the vertebral mesh. A partial-thickness trough was created on the contralateral side to simulate a hinge at the lamina-facet junction, as is standard in open-door laminoplasty procedures [15,16]. Furthermore, the ISL and LF were disconnected at the C3–C4 and C6–C7 junctions. Subsequently, the laminae of C4, C5, and C6 were rotated between 13° and 16° towards the created hinge, replicating the surgical maneuver to elevate and hold the laminae in an open position. A titanium plate was installed to maintain the laminoplasty’s integrity, with the model using hexahedral elements to represent the plate accurately using the ANSA^®^ v22.1.0 (BETA CAE Systems, Farmington Hills, MI, USA) software The 1 mm thick titanium plate with Young’s modulus of 110 Gpa and Passion’s ratio of 0.3, modeled after the ARCH laminoplasty system from DePuy Synthes, was contoured to ensure an optimal fit. Tight contact conditions were established at the interface between the plate and the vertebral elements (lateral mass and lamina). Furthermore, the spinal cord was repositioned dorsally by an average of 1 mm at the decompressed levels, simulating the anticipated spinal cord shift after laminoplasty, and increasing the cerebrospinal fluid volume in the space generated by the laminoplasty [17].

### 2.6. Loading and Boundary Conditions

The biomechanical responses of the spinal column and spinal cord were evaluated during flexion and extension, utilizing LS-PrePost v4.3 and LS-Dyna v10.2.0 (Livermore, CA, USA). In the loading conditions, a consistent moment of 2 Nm was applied in both flexion and extension [10]. Additionally, a follower load of 75 N was implemented during flexion and extension to realistically represent the impact of head mass and cervical neck muscles [18,19]. The models were fixed at the inferior surface of the T1 vertebra, restricting movement in all six degrees of freedom. The parameters measured included the segmental range of motion (ROM), spinal cord stress (von Mises Stress), and strain (Maximum Principal Strain). To ensure data reliability, the average stress and strain values recorded were processed to omit any data falling below the 5th percentile or above the 95th percentile [20].

## 3. Results

### 3.1. Segmental Range of Motion

**Figure 3:**
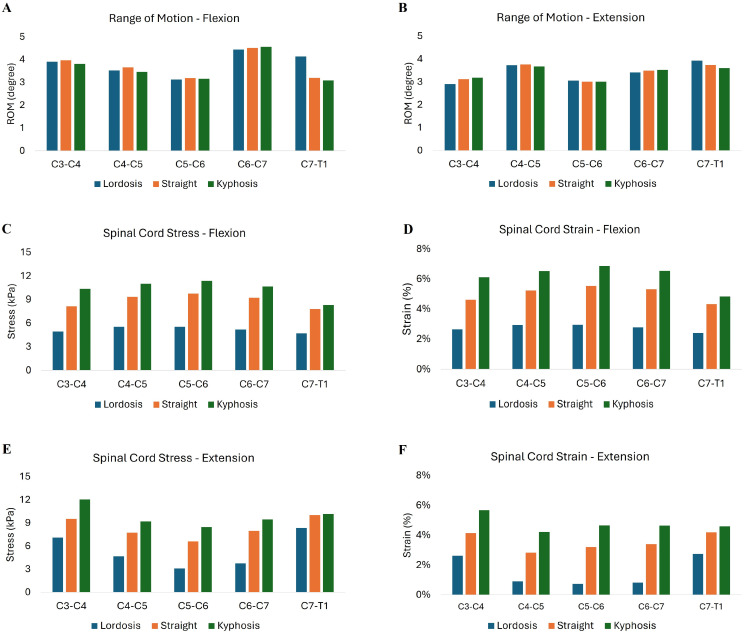
Segmental range of motion (ROM) during flexion (**A**) and extension (**B**) for lordotic, straight, and kyphotic sagittal spine alignments after cervical laminoplasty. Spinal cord stress (**C**,**E**) and strain (**D**,**F**) during neck flexion and extension are shown.

The segmental ROM was analyzed at the levels of laminoplasty (C4–C5, C5–C6, and C6–C7) across different sagittal alignments (lordotic, straight, and kyphotic) during both flexion and extension. The specific spinal alignment does not impact the segmental ROM at the surgical level. In flexion, the mean ROM for the lordotic spine at the laminoplasty levels was 3.70 degrees (SD 0.57); for the straight spine, it was 3.78 degrees (SD 0.57); and for the kyphotic spine, it was 3.73 degrees (SD 0.81) (Figure 3A). During extension, the mean ROM for the lordotic spine at these levels was 3.40 degrees (SD 0.32); for the straight spine, it was 3.43 degrees (SD 0.42); and for the kyphotic spine, it was 3.40 degrees (SD 0.41) (Figure 3B).

### 3.2. Spinal Cord Stress and Strain at Decompressed Levels

Spinal cord stress and strain was highest for kyphotic curvature compared to straight and lordotic curvature across all decompressed levels. When the kyphotic spine was in flexion, the average spinal cord stress from C4–C7 was 103.3% higher than the lordotic spine and 16.7% higher than in the straight spine (Figure 3C). The spinal cord strain in the kyphotic spine in flexion was 128.9% higher than the lordotic spine and 26.8% higher than the straight spine (Figure 3D). Similarly, spinal cord stress during neck extension in the kyphotic spine was 135.4% higher than the lordotic spine and 21.5% higher than the straight spine (Figure 3E). Spinal cord strain in the kyphotic spine during neck extension was 241.7% higher than the lordotic spine and 43.2% higher than the straight spine (Figure 3F).

### 3.3. Spinal Cord Stress and Strain at Adjacent Segments

**Figure 4:**
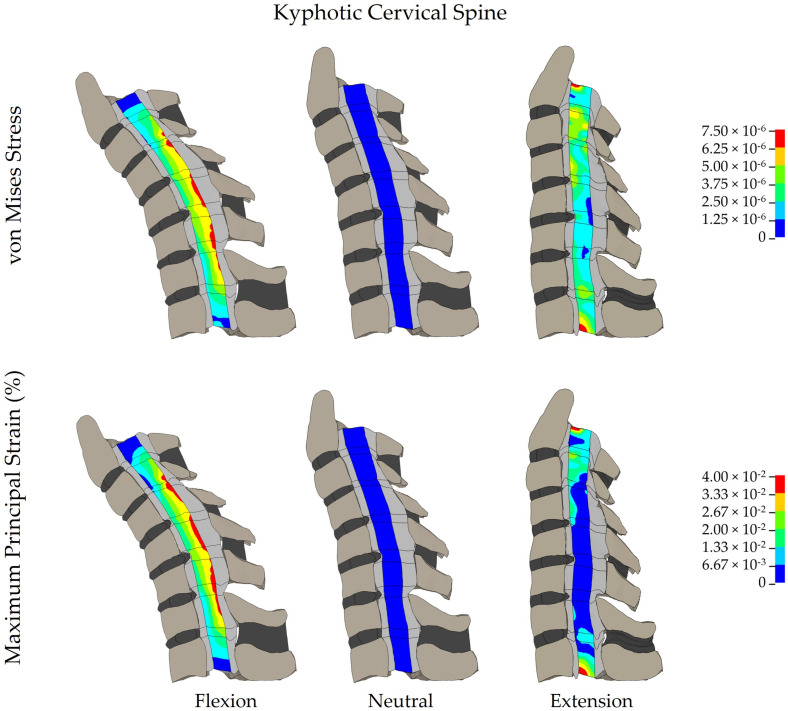
Distribution of spinal cord stress and strain in a kyphotic cervical spine.

Spinal cord stress and strain were increased in the kyphotic spine at both superior (C3–C4) and inferior (C7–T1) adjacent segments (Figure 4) when compared to the lordotic and straight spines. The magnitude of the difference in spinal cord stress and strain at the adjacent segments was greater for neck flexion compared to neck extension in all three models. When the kyphotic spine was flexed, the spinal cord stress at the superior adjacent level was 109.9% higher than the lordotic spine in flexion and 27.4% higher than in the straight spine. The spinal cord strain at the superior level of the kyphotic spine in flexion showed a 131.3% compared to the lordotic spine and 32.4% higher compared to the straight spine. During extension at the superior adjacent level, the spinal cord stress in the kyphotic spine increased by 69.7% compared to the lordotic spine and 26.2% compared to the straight spine. The spinal cord strain during extension at the superior level was 116.7% greater in the kyphotic spine compared to the lordotic spine and 37.3% higher than in the straight spine. Similar differences in spinal cord stress and strain were observed in the kyphotic spine at the inferior (C7–T1) adjacent segment; however, the magnitude of the increase in spinal cord stress and strain was lower than the superior adjacent segment.

### 3.4. Effect of Increasing Kyphosis on Spinal Cord Stress and Strain

For every one degree increase in kyphosis after laminoplasty, the overall spinal cord stress increased by 0.35 kPa, and the spinal cord strain increased by 0.21% when the cervical spine was flexed. Similarly, for every one degree increase in kyphosis after laminoplasty, spinal cord stress increased by 0.36 kPa, and spinal cord strain increased by 0.42% when the cervical spine was extended.

## 4. Discussion

This study used a finite element model of the C2–T1 cervical spine and spinal cord to evaluate the impact of sagittal alignment on spinal cord biomechanics after cervical laminoplasty. Our model demonstrated that cervical kyphosis is associated with elevated spinal cord stress and strain after laminoplasty, both at the decompressed and adjacent segments. The adverse effect of cervical kyphosis on spinal cord biomechanics was noted despite the similar segmental range of motion and dorsal spinal cord shift for the kyphotic, lordotic, and straight alignments.

Cervical laminoplasty is a motion-preserving intervention for DCM [21], and sagittal alignment of the cervical spine has been shown to affect the outcomes after laminoplasty. Local kyphosis exceeding 13° is a risk factor for suboptimal recovery for DCM patients undergoing laminoplasty [3]. The K-line, or the line connecting the midpoints of the spinal canal at C2 and C7, has also been used to predict postsurgical outcomes and residual cord compression after cervical laminoplasty [22,23]. In addition, kyphosis can develop after laminoplasty, and the key predictors of post-surgical kyphosis include age, BMI, preoperative flexion and extension of C2–C7 segments, and sagittal vertical axis [24,25]. This study identified the effect of cervical kyphosis on spinal cord biomechanics, which further explains the effects of kyphosis on neurological outcomes. These results are expected to help surgeons better predict neurological recovery after laminoplasty based on the pre-surgical sagittal alignment.

Finite element models are an increasingly effective surgical tool to analyze the biomechanics of the spinal cord after surgical intervention. Prior FEM studies have shown variations in spinal cord stress and strain after laminectomy, laminectomy with fusion, or open-door laminoplasty [1,8]. Although laminoplasty is a motion-preserving surgery, prior clinical studies [26] have shown a 20% reduction in neck range of motion after laminoplasty, which could lead to increased compensatory range of motion at the adjacent segments. To support this clinical finding, we found that range of motion and spinal cord stress and strain were increased at the adjacent segments, especially in the kyphotic alignment.

The results of this study support prior work showing that loss of sagittal alignment in the cervical spine is associated with poorer neurological outcomes in DCM [27,28]. The clinical implication of this study is that addressing spinal alignment in addition to spinal cord decompression is necessary to optimize spinal cord biomechanics after laminoplasty. Patients with preoperative kyphosis are not ideal candidates for cervical laminoplasty since the surgical approach cannot correct the sagittal alignment. These patients are better served by an anterior cervical approach for decompression and restoration of lordosis to optimize neurological outcomes. While physiotherapy can strengthen the paraspinal muscles preoperatively, it is unlikely to provide durable correction of sagittal alignment [29]. Therefore, preoperative assessment of sagittal alignment should be incorporated into surgical decision making to optimize neurological outcomes. This study provides a biomechanical basis for poorer neurological outcomes for those patients in whom the sagittal alignment is not addressed when performing a cervical laminoplasty. The development of patient-specific FEMs could further help with clinical decision-making in the future. By incorporating patient-specific geometries, surgeons can use the FEM method to estimate the degree of lordosis correction required to reduce spinal cord stress/strain for the individual patient. Additionally, surgeons could use the FEM method to predict biomechanical outputs for different surgical approaches for the individual patient [1].

This study has several limitations. We used a modified generic model of the cervical spine and spinal cord with different sagittal alignments, and the results may not apply to patients with kyphosis or lordosis that is outside the range studied. Tissue-specific properties of the spinal cord gray and white matter are not available and were not incorporated into the model. The effect of spondylolisthesis as well as facet arthropathy will need to be evaluated in future studies. In this study, we used a single laminoplasty technique (open-door laminoplasty) for our surgical simulations, but the FEM method can be used to quantify the effect of spinal alignment on spinal cord biomechanics after other laminoplasty techniques such as double-door laminoplasty [30].

## 5. Conclusions

Finite element analysis of spinal cord biomechanics showed considerably greater spinal cord stress and strain after cervical laminoplasty in the kyphotic spine. These biomechanical insights, supported by clinical evidence from previous studies, can guide surgeons towards addressing sagittal alignment in addition to spinal cord decompression for DCM.

## Data Availability

The data presented in this study are available on request from the corresponding author.
